# A Randomised Controlled Trial Comparing Induction of Labour with the Propess Vaginal System to the Prostin Vaginal Tablet in Premature Rupture of Membranes at Term

**DOI:** 10.3390/jcm12010174

**Published:** 2022-12-26

**Authors:** Veronika Anzeljc, Faris Mujezinović

**Affiliations:** 1 Department of Perinatology, University Medical Centre Maribor, Ljubljanska 5, 2000 Maribor, Slovenia; 2 Faculty of Medicine, University of Maribor, Taborska 8, 2000 Maribor, Slovenia

**Keywords:** premature rupture of membranes at term, induction of labour, a randomised controlled trial, propess vaginal system, prostin vaginal tablet

## Abstract

**Aim**: To compare the perinatal outcome and delivery intervals after the induction of labour with the Prostin vaginal tablet versus the Propess vaginal system in pregnant women with term-PROM. **Design**: One centre paralleled randomised controlled trial with a computer-generated table to allocate treatments. **Setting**: University Medical Centre in Slovenia. **Participants**: A total of 205 singleton healthy pregnant women with term-PROM. **Intervention**: Induction of labour with the Propess vaginal system (intervention group) versus Prostin tablets (control group). **Main outcomes**: The rate of failed inductions, complications in labour, time intervals between the PROM, induction, the beginning of the active phase, and delivery. **Results**: A total of 104 patients received Prostin, and 101 patients received Propess. Induction failure rates in the Prostin and the Propess groups were 8/104 (7.7%) and 5/101 (5.0%), respectively (*p* = 0.80). Delivery abnormalities were uncommon and comparable across the groups. The rates of caesarean sections in the Prostin and Propess groups were 4/96 (4.2%) and 6/96 (6.3%), respectively. The delivery intervals were comparable across the groups. **Conclusions**: In term-PROM pregnancies, the Propess vaginal system is a safe and effective option for inducing labour.

## 1. Introduction

The premature rupture of membranes (PROM) refers to amniotic fluid leakage from the vagina near the expected date of delivery [1]. It is a common obstetric event that occurs in 8–10% of pregnancies [1]. This is usually followed by labour and delivery, which can happen spontaneously or with the help of various methods for inducing labour [2]. Which option represents the best medical practice in terms of a waiting period or labour induction method is still a matter of debate [3].

The decision is simpler in the case of a favourable cervix, where oxytocin is used to establish proper uterine contractions and accelerate the active stage of labour [1]. However, if the cervix is unfavourable (Bishop 6 or less), PROM will necessitate cervical ripening, which is similar to labour induction without PROM and is usually initiated with dinoprostone [4].

An unfavourable cervix in term-PROM brings up new issues that have been highlighted in recent years [4]. One of them is the decision of whether or not to wait for the cervix to mature naturally. Another strategy involves using medications for labour induction more proactively. When to use medicine to advance labour, how to improve women’s satisfaction with their overall delivery experience, and prevent intrauterine infections are other crucial factors to take into account [3]. The ease of administration, effectiveness, and safety profiles of the medications or devices that are available for labour induction in term-PROM heavily influence this dilemma.

A new vaginal device (Propess^®^) (Ferring Pharmaceuticals, Saint-Prex, Switzerland) with a slow and controlled release of dinoprostone has been introduced into clinical practice in recent years, simplifying the induction process [5]. This system contains 10 mg of PGE2, which is released at a rate of 0.3 mg/h over a 24-hour period. This reduces the need for frequent vaginal examinations and tablet insertion, as well as the risk of infection [6].

This modality is expected to have numerous advantages over standard prostaglandin tablets [6]. However, the Propess manufacturer states that PROM is one of the medication’s contraindications due to a lack of evidence on its safety and efficacy [5,7].

As a result, we completed a randomised controlled trial in pregnant women with term-PROM, comparing the efficacy and safety of prostaglandin tablets and an intravaginal device.

## 2. Materials and Methods

### 2.1. Enrollment of Patients

Pregnant women at term (37–41 weeks of pregnancy) with PROM and an unfavourable cervix with no contractions were invited to participate in our study. From 11 March 2021 to 31 August 2022, the study was conducted at the Department of Perinatology, University Medical Centre Maribor, Slovenia. PROM was identified by vaginal inspection following amniotic fluid leakage via the cervical orifice and accumulation in the posterior vaginal fornix or by a positive test for the protein insulin-like growth factor-binding protein 1 (IGFBP-1) in the vaginal fluid. All patients who participated in the study signed an informed consent form. The criteria for inclusion in the study were: age 18 years or older, singleton pregnancy, cephalic insertion of the foetus, no signs of chorioamnionitis, no previous surgery on the uterus, no contraindications for vaginal delivery, absence of uterine contractions, and unfavourable cervix (defined as a Bishop score of six points or less) at least 4 h after PROM. In patients with PROM and meconium-stained amniotic fluid but normal cardiotocography (CTG) tracing, we started the induction immediately upon arrival in the delivery room.

We excluded pregnant women who met the following criteria: age less than 18 years old, previous uterine surgery (previous caesarean sections, myomectomies), multiple pregnancies, a Bishop score of seven points or higher, signs of chorioamnionitis, elevated body temperature or other signs of inflammation at the time of admission to the Department of Perinatology, an inconclusive or pathological cardiotocographic (CTG) record, and all non-cephalic pregnant women who had a positive Streptococcus B vaginal smear or urine test were given penicillin-based antibiotics on a regular basis as soon as they were admitted to the delivery room. Others were given antibiotics 8 h after PROM, in accordance with standard hospital clinical policy.

### 2.2. Random Allocation

Pregnant women with term-PROM were individually and randomly assigned to receive either vaginal Prostin tablets or a Propess vaginal insert (parallel design). A computer randomization process with a 1:1 allocation ratio was used to assign patients to study groups. Initially, our technical staff generated a randomisation list using the established tool (simple randomiser) at https://www.sealedenvelope.com/simple-randomiser/v1/lists (accessed on 5 January 2021) with block sizes of four, six, and eight. The generated list was imported into Google Sheets, and a Google Apps script was created to generate a PDF file containing the allocation information from this table for each patient. The Google Apps script was triggered by the submission of a Google form.

The women’s information was entered into a Google form by the doctor treating the patient, and the software assigned them to a group. Nobody knew which study group the pregnant women would be assigned to before the computer assigned them to a specific group. Because medication concealment was not possible after allocation, all study participants were aware of the patient study allocation. All outcomes of the delivery were documented in the patient’s official medical record, as required by law and valid in all Slovenian hospitals.

### 2.3. Induction of Labour

Following study enrolment, deliveries were managed by a midwife and a gynaecology and obstetrics specialist in accordance with the established clinical guidelines and professional rules. In the intervention group, the Propess 10 mg vaginal system was inserted into the posterior vault of the vagina with the examiner’s fingers while the patient was lying on a bed in the delivery room. The Propess 10 mg vaginal system was left in place for up to 24 h. We removed it from the vagina earlier when the cervical dilation was 3–4 cm, there were regular uterine contractions, and the active phase of labour had begun. We monitored the CTG for at least an hour after Propess insertion.

In the control group, the Prostin 3 mg vaginal tablet (Pfizer, New York, NY, USA) was inserted with the examiner’s fingers high into the fornix of the vagina while the patient was in a lying position in a bed in the delivery room. In the case of a persistently unfavourable cervical condition, two additional doses were administered eight hours apart. CTG monitored the foetus’ heartbeats for at least an hour. CTG monitoring was extended beyond one hour in cases of unconvincing CTG tracing.

The management of PROM in cases with an unresponsive cervix, even 24 h after induction, was left to the expert judgement of the supervising obstetrician and the pregnant woman’s wishes in terms of a new induction cycle or a caesarean section.

### 2.4. Study Outcomes

The study’s objectives were to compare the rates of failed inductions and delivery complications between the groups, as well as the time intervals between the PROM, induction, active phase starts, and delivery. The induction active phase (IA) interval was measured in minutes and denoted the time between the induction and the start of the active phase, which was defined as a cervical dilatation of 6 cm. For the control group (Prostin 3 mg vaginal tablets) and the experimental group (Propess 10 mg vaginal system), the expected average IA intervals were 300 min (5 h) and 240 min (4 h), with a standard deviation of 60 min, respectively. To find such a difference, we needed a study sample of 63 patients in each group, for a total of 126 patients in the study.

The induction delivery (ID) interval was measured in minutes and described the time between the induction and the delivery. The PROM delivery (PD) interval was expressed in minutes and described the time between the PROM and the delivery. The PROM induction (PI) interval was expressed in minutes and described the time between the PROM and the induction. The active phase delivery (AD) interval (in minutes) describes the time between the start of the active phase and the time of delivery.

### 2.5. Sample Size Estimation

To determine a 15% difference in complications, we needed at least 97 pregnant women in each group, and a total of 194 pregnant women for the entire study, for an expected 25% failure rate in the control group and a 10% failure rate in the experimental group (failed inductions, caesarean sections). For categorical variables, we used the binary outcome superiority trial calculator, and for relational variables, we used the continuous outcome superiority trial calculator (https://www.sealedenvelope.com/power/continuous-superiority/, accessed on 5 January 2021) [8].

**Figure 1:**
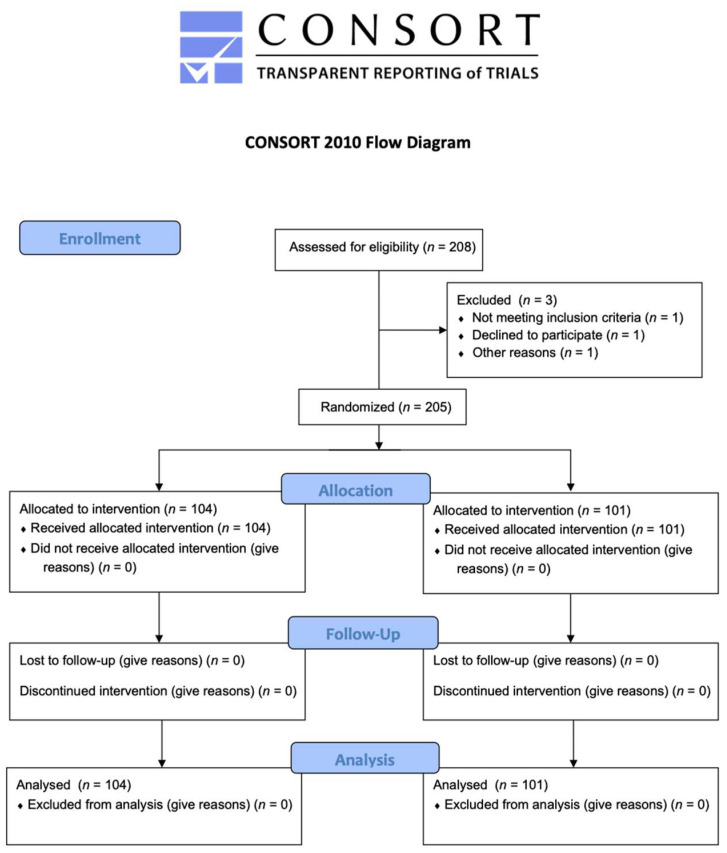
Consort flow chart of the selection process of the patients studied. IoL—induction of labour, PROM—premature rupture of membranes.

As a result of the foregoing, we enrolled 200 pregnant women in the research study (100 pregnant women in each group). A power level (1-beta) of 80% and a significance level of alpha at 5% were chosen. The study was approved by the Republic of Slovenia’s Medical Ethics Commission (Number: 0120-35/2021/8). On 2 February 2021, the trial was registered at clinicaltrials.gov (identification number: NCT04743297). The CONSORT statement (http://www.consort-statement.org, accessed on 5 January 2021) and clinical study conduct and publication standards were followed in our study. The results are shown in Figure 1.

### 2.6. Statistical Analysis

R (a programming language for statistical computing and graphics) was used for statistical analysis [9]. The population’s characteristics were represented as continuous or categorical variables and were calculated as frequencies or means (standard deviations (SD)). The chi-square test was used to compare the categorical variables. The Mann–Whitney U-test or *t*-test (depending on the normality of the distribution of values) was used for the continuous variables. The impact of various parameters on the various perinatal outcomes investigated was then investigated using bivariate and multivariate analyses. Multiple logistic or multiple linear regression was used depending on whether the outcome variable was categorical or quantitative. At *p* < 0.05, statistically significant differences were found.

## 3. Results

We included 205 pregnant women in our study. Among them, 104 and 101 pregnant women were assigned to the Prostin and Propess groups, respectively. There were no statistically significant differences in demographic characteristics between the two groups. Table 1 provides a more detailed insight into the demographic data.

Table 2 contains details about the induction. There were no repetitions of the induction process in the study. Induction failure rates for the Prostin and Propess groups were 7.7% and 5.0%, respectively. The difference in failure rates between the groups was not statistically significant.

Table 3 shows that the duration of labour and delivery was comparable between the groups on average.

As shown in Table 4, delivery abnormalities were uncommon and comparable across both groups. The rates of caesarean sections in the Prostin and Propess groups were 4.2% and 6.3%, respectively. The rates of vacuum extraction in the Prostin and Propess groups were 10.4% and 11.5%, respectively. There were no statistically significant differences in neonatal data between the groups (Table 5).

A comparison of different intervals between the key moments in delivery management, such as the time of PROM, induction, the start of the active phase, and the time of the baby’s birth, revealed no differences between the research groups (Table 6a). However, for the entire study cohort as well as for the Prostin group, the subgroup of patients with a more unfavourable Bishop score (three or less) had a statistically significant longer induction-active phase (IA), PROM-delivery (PD), and induction-delivery (ID) intervals (Table 6b). The influence of the Bishop score on observed intervals in the Propess group was less pronounced and statistically significant only for the IA phase (Table 6b).

The correlation between the IA interval and the PD interval was significant (Spearman correlation coefficient 0.83, *p* < 0.001), as was the correlation between the IA interval and the ID interval (Spearman correlation coefficient 0.98, *p* < 0.001) (Table 7).

The risk of operative delivery was associated with primiparous women, according to multiple logistic regression (aOR 5.81, (95% CI 1.68–20.11), *p* = 0.0053) (Table 8). According to a linear regression model, the ID interval was related to the IA interval (estimate 1.022, *p* < 0.0001) and was longer in primiparous women (coefficient = 81.611, *p* < 0.001, multiple R squared 0.976, *p* < 0.001). The multiple linear regression model revealed that the AD interval was only predicted by the woman’s primiparous status (coefficient = 81.611, *p* < 0.001). R squared multiple 0.244, *p* = 0.001 (Table 8).

## 4. Discussion

### 4.1. Principal Findings

In terms of safety, our randomised controlled trial found that the Propess vaginal device had a low rate of complications, comparable to Prostin tablets: the standard method of cervical ripening and induction in PROM in near-term pregnancies. In our study, 11.5% and 10.9% of the Prostin and Propess groups had total caesarean sections, respectively, and 10.4% and 11.5% had vacuum extraction. There were no statistically significant differences in operative deliveries or neonatal data.

Failed induction occurred in 13 cases (6.4%) of our cohort of patients, resulting in operative deliveries, with no difference in the distribution across groups (7.7% in the Prostin group and 5.0% in the Propess group, *p* = 0.80). There was no statistical difference in the caesarean section rate during the active phase of delivery between the Prostin and Propess groups. Overall, the number of caesarean deliveries was low, which was consistent with our estimate of the control group’s caesarean section rate (10%). It is worth noting that none of the pregnant women with PROM wanted to wait for their own contractions and spontaneous labour.

The primary goal of this study was to compare the individual intervals between PROM and the baby’s birth. All-important intervals were comparable across both groups, including the PI interval, IA interval, AD interval, PD interval, and ID interval. According to subgroup analysis, the IA interval, PD interval, and ID interval were all longer in the group with more unprepared cervixes for dilatation and delivery (the three or less subgroup and the four or more group). There was a strong correlation (*p* < 0.0001) between the IA interval and the PD interval, as well as between the IA interval and the ID interval. We also wanted to find potential predictors of caesarean section (CS) in cases of vaginal dinoprostone induction in term-PROM.

Multiple linear regression revealed that the pregnant women’s primiparous status (*p* < 0.0001) and the length of the interval between induction and the start of the active phase of delivery were the factors that most influenced the ID interval. Multiple logistic regression revealed that the pregnant women’s primiparous status increased the odds ratio (aOR 5.81) for operative delivery and that the Bishop score was merely a confounding factor.

### 4.2. Results in the Context of What Is Known

Despite the fact that vaginal dinoprostone is the preferred method of induction in labour in term-PROM, no studies have directly compared the safety and efficacy of Prostin tablets to vaginal inserts, as we have conducted in our study [10]. There have been some studies showing that the induction of labour in term-PROM pregnancies is as safe as induction in non-PROM pregnancies [11].

The caesarean section rate in our study is significantly lower than that of other studies that reported their findings in a variety of settings. López-Jiménez, for example, found that the PROM group (28.7%) had a similar caesarean rate to the non-PROM group (34.2%) [11]. A c-section rate of 21.6% was also reported for the Prostin group in a study by Liu et al., which compared the safety and efficacy of Prostin alone and Prostin in combination with oxytocin [12]. Other studies comparing vaginal dinoprostone with oxytocin in term-PROM pregnancies found a similar success rate. In Rijal et al., a randomised study from Nepal, the vaginal gel Prostin group had an 88.8% success rate of induction. The success rate of induction was comparable to that of oxytocin but with longer induction to delivery intervals. The study included 72 patients [13].

Higher caesarean section rates were also reported in a study by Kunt et al. that compared the effect of oxytocin and vaginal Prostin tablets in pregnant women with term-PROM 12 h after PROM. The oxytocin and Prostin groups had statistically similar Caesarean delivery rates (18.3 vs. 20.0%; *p* = 0.81) [14]. We discovered that primiparous women had a higher chance of having a c-section, but Sobande et al. discovered no differences in induction success between primiparous and multiparous women [15].

The average induction to delivery interval for Wang’s PROM group was shorter than for the non-PROM group, but it was still 1125 min. This is 317 min longer than the average of our study cohort [16]. Kehl et al., on the other hand, reported PROM group induction to delivery intervals that were closer to ours (972 min) [17]. Similarly, López-Jiménez et al. reported a total duration induction of 1884 min in the PROM group, which is significantly longer than our induction to active phase time, which averaged 670 min in both groups [11]. It is difficult to determine the causes of such disparities.

### 4.3. Clinical Implications

Our findings suggest that the Propess vaginal device can be used safely during the induction process in pregnancies with term-PROM, and this should be stated in the Propess vaginal device’s formal instructions. Labour induction in term-PROM is effective and does not necessitate multiple repetitions of the induction protocol.

Our findings also raise the question of whether the number of caesarean sections would be reduced, particularly during the induction phase, if the interval between PROM and induction was extended and more women gave birth naturally without the use of prostaglandins. Larger controlled trials have already confirmed these assumptions [18].

However, new evidence suggests that inducing labour immediately in term-PROM pregnancies is the best management strategy for minimising neonatal and maternal morbidity [19].

### 4.4. Research Implications

More research on women’s preferences, which can influence the final choice of medication, should be conducted, even in different forms of the same drug, as is the case with Prostin tablets and the Propess vaginal device, both of which contain dinoprostone, because unnecessary vaginal examinations can be distressing for women [20].

Our findings indicate that the Bishop score and the woman’s primiparous status may influence the outcome of induction in term-PROM pregnancies, and we believe that these factors should be researched further in the future.

### 4.5. Strengths and Limitations

The randomised prospective design of our study allows for the effect of medications to be more reliably assessed. We used well-defined and agreed-upon variables, as well as a consistent induction protocol. The sample sizes in our study groups were large enough to detect clinically significant differences. Furthermore, given the scarcity of evidence on the use of various forms of dinoprostone for PROM, we believe that the findings of this study could help improve the performance of meta-analyses with greater statistical power in order to establish firm recommendations on the most appropriate method of cervical ripening and induction in this group of patients.

One limitation of our study was the small number of patients used to compare rare adverse events such as uterine rupture or admission to the neonatal intensive care unit.

## 5. Conclusions

To summarise, our study discovered that the safety and efficacy of various intervals between induction and delivery were comparable across the study groups. We have demonstrated that an intravaginal induction device can be used safely in pregnant women with term-PROM, raising the question of how to improve the chances of successful vaginal delivery in patients with lower Bishop scores, particularly in primiparous women.

## Figures and Tables

**Table 1:** Demographic characteristics of the study population according to the study groups. SGB—streptococcus group B, SD—standard deviation, N—number of cases, %—percentage of the group.

Parameter	Prostin (*n* = 104)		Propess (*n* = 101)		Total (*n* = 205)		*p* Value
	Average	SD	Average	SD	Average	SD	
Age (years)	30.4	4.3	31.1	5.2	30.7	4.7	0.36
Height (cm)	167.3	5.6	166.3	6.4	166.8	6.0	0.24
Weight at conception (kg)	68.4	14.4	66.1	15.0	67.3	14.7	0.25
Weight at delivery (kg)	83.3	14.2	80.3	16.2	81.8	15.2	0.17
	**N**	**%**	**N**	**%**	**N**	**%**	
Number of pregnancies							
One	60	57.7	56	55.5	116	56.6	0.85
Two and more	44	42.3	45	44.5	89	43.1	
Number of deliveries							
One	74	71.2	73	72.3	147	71.7	0.98
Two or more	30	28.9	28	27.7	58	28.3	
Smoking (Yes)	7	6.7	5	5.0	12	5.9	0.81
Disease before pregnancies							
Yes	10	9.6	8	7.9	18	8.8	0.86
No	94	90.4	93	92.1	187	91.2	
Diseases in pregnancies							
Yes	62	59.6	63	62.4	125	61.0	0.79
No	42	40.4	38	37.6	80	39.0	
SGB positive							
Yes	5	4.8	10	9.9	15	7.3	0.26
No	99	95.2	91	90.1	190	92.7	
Days in hospital							
4 or less	74	71.2	71	70.3	145	70.7	1.0
5 or more	30	28.8	30	29.7	60	29.3	
Weeks of gestation							
37	13	12.5	10	9.9	23	11.2	0.36
38	18	17.3	26	25.7	44	21.5	
39	28	26.9	32	31.7	60	29.3	
40	37	35.6	29	28.7	66	32.2	
41	8	7.7	4	3.96	12	5.85	
Bishop score at induction of labour							
From 1 to 3	35	33.6	45	44.6	80	39.1	0.15
From 4 to 6	69	66.4	56	55.4	125	60.9	

**Table 2:** Induction data of the study population according to the study groups. N—number of cases, %—percentage of the group, CI—confidence interval.

Parameter	Prostin (*n* = 104)		Propess (*n* = 101)		Total (*n* = 205)		*p* Value	Effect Size (CI)
	N	%	N	%	N	%		
Number of repetitions								
1	69	66.3	101	100.0				
2	21	20.2	0	0.0				
3	14	13.5	0	0.0				
Propess usage duration								
12 h or less			77	76.2				
13 h and more			24	23.8				
24 h pause								
No	104	100	101	100	205	100.0	1.00	
Yes	0	0.0	0	0.0	0	0.0		
Failed induction								
No	96	92.3	96	95.0	192	93.6	0.80	1.03
Yes	8	7.7	5	5.0	13	6.4		(0.96–1.11)
Cause of failed induction								
Unresponsive cervix	3	37.5	0	0.0	3	23.1	0.38	
Pathologic CTG	5	62.5	5	100	10	76.9		
Total	8	100.0	5	100.0	13	100.0		

**Table 3:** Delivery data of the study population according to the study groups. SD—standard deviation, N—number of cases, %—percentage of the group, CI—confidence interval.

Parameter	Prostin (*n* = 96)		Propess (*n* = 96)		Total (*n* = 192)		*p*	Effect Size (CI)
	Average	SD	Average	SD	Average	SD		
Oxytocin max. dosage	9.1	5.0	9.2	5.3	9.4	4.9	0.47	0.02 (−0.26–0.30)
	**N**	**%**	**N**	**%**	**N**	**%**		
Oxytocin usage								
No	37	38.5	35	36.5	72	37.5	0.96	
First stage	35	36.5	36	37.5	71	37.0		1.00 (0.69–1.45)
Second stage	24	25.0	25	26.0	49	25.5		1.04 (0.64–1.68)
Fetal scalp sampling								
No	83	86.5	83	87.5	167	87.0	1.0	
Yes	13	13.5	13	12.5	26	13.5		1.00 (0.89–1.12)
pH level—fetal scalp blood sampling								
Equal and less then 7.24	9	69.2	7	58.3	16	61.5	0.67	0.78 (0.30–2.00)
7.25 and more	4	30.8	6	50.0	10	38.5		
Fetal leading part								
Occipit Anterior	94	97.9	94	97.9	188	97.9	0.14	
Occipit Posterior	2	2.1	2	2.1	4	2.1		1.00 (0.14–6.96)
Labor analgesia								
No	7	7.3	7	7.3	14	7.3	0.26	1.00 (0.36–2.74)
Epidural	42	43.8	53	55.2	95	49.5		
Intravenous	47	49.0	36	37.5	83	43.2		

**Table 4:** Delivery abnormalities data of the study population according to the study groups. N—number of cases, %—percentage of the group, CI—confidence interval.

Parameter	Prostin (*n* = 96)		Propess (*n* = 96)		Total (*n* = 192)		*p*	Effect Size (CI)
	N	%	N	%	N	%		
Delivery abnormalities								
No	79	82.3	77	80.2	156	81.3	0.85	
Yes	17	17.7	19	19.8	36	18.8		1.1 (0.6–2.0)
Operative delivery								
No	82	85.4	79	82.3	161	83.9	0.78	
Caesarean section	4	4.2	6	6.3	10	5.2		1.5 (0.4–5.2)
Vacuum extraction	10	10.4	11	11.5	21	10.9		1.1 (0.5–2.5)
Cervical dilatation in time of caesarean section (cm)								
6 or less	2	50.0	4	66.7	6	60.0	0.78	
7 or more	2	50.0	2	18.2	4	40.0		
Episiotomy								
No	61	63.5	56	58.3	117	60.9	0.55	
Mediolateral	35	36.5	40	41.7	75	39.1		1.1 (0.8-1.6)
Rupture								
No	63	65.6	58	60.4	121	63.0	0.75	
Small vaginal rupture	31	32.3	36	37.5	67	34.9		1.2 (0.8–1.7)
III- and IV-degree perineal rupture	2	2.1	2	2.1	4	2.1		1.0 (0.1–7.0)

**Table 5:** Neonatal data of the study population according to the study groups. N—number of cases, %—percentage of the group., CI—confidence interval.

Parameter	Prostin (*n* = 104)		Propess (*n* = 101)		Total (*n* = 205)		*p* Value	Effect Size (CI)
	N	%	N	%	N	%		
Apgar in 1. min								
7 and lower	9	8.7	9	8.9	18	8.8	1.0	1.0 (0.4–2.5)
8 and higher	95	91.3	92	91.1	187	91.2		
Apgar in 5. min								
7 and lower	3	2.9	3	2.97	6	2.93	1.0	1.0 (0.2–5.0)
8 and higher	101	97.1	98	97.03	199	97.07		
Apgar in 10. min								
7 and lower	0	0.0	2	1.98	2	0.98	0.46	5.1 (0.3–105.9)
8 and higher	104	100.0	99	98.02	203	98.02		
Admission to intensive care unit								
No	104	100.0	100	99.01	204	99.5	0.89	
Yes	0	100.0	1	0.99	1	0.5		3.1 (0.1–74.9)

**Table 6:** Individual delivery interval lengths across different criteria. SD—standard deviation, CI confidence interval.

(a) Mean values and standard deviations of the individual delivery interval lengths compared across research groups.									
**Parameter**	**Prostin (*n* = 96)**		**Propess (*n* = 96)**		**Total (*n* = 192)**		***p*** **Value**	**Effect Size (CI)**	
	Average	SD	Average	SD	Average	SD			
PROM—Induction (min)	480	395	473	323	476	360	0.94	−0.02 (−0.3–0.3)	
	Prostin (*n* = 94)		Propess (*n* = 92)		Total (*n* = 186)				
	Average	SD	Average	SD	Average	SD			
Induction—Active phase (min)	683	413	657	486	670	450	0.70	−0.06 (−0.3–0.2)	
	Prostin (*n* = 92)		Propess (*n* = 90)		Total (*n* = 182)				
	Average	SD	Average	SD	Average	SD			
Active phase—Delivery (min)	128	89	138	79	133	84	0.43	0.12 (−0.17–0.41)	
	Prostin (*n* = 92)		Propess (*n* = 90)		Total (*n* = 182)				
	Average	SD	Average	SD	Average	SD			
PROM—Delivery (min)	1299	577	1252	607	1276	590	0.60	−0.08 (−0.4–0.2)	
	Prostin (*n* = 92)		Propess (*n* = 90)		Total (*n* = 182)				
	Average	SD	Average	SD	Average	SD			
Induction—Delivery (min)	817	440	799	519	808	479	0.80	−0.04 (−0.32–0.25)	
(b) Mean values and standard deviations of the individual delivery interval lengths compared across research groups by Bishop score.									
**Parameter**	**Group**	**Bishop Three or Less**			**Bishop Four or More**		**Bishop (*n* = 192)**	***p*** **Value**	
		Average		SD	Average	SD	Average	SD	
PROM—Induction (min)	All (*n* = 192)	467		310	481	388	476	360	0.58
	Prostin (*n* = 96)	476		307	481	434	480	395	0.61
	Propess (*n* = 96)	459		316	482	331	472	323	0.91
		Average		SD	Average	SD	Average	SD	
Induction—Active phase (min)	All (*n* = 186)	853		511	563	372	670	450	0.0001
	Prostin (*n* = 94)	947		458	559	327	683	413	0.0001
	Propess (*n* = 92)	780		422	566	422	657	487	0.04
		Average		SD	Average	SD	Average	SD	
Active phase—Delivery (min)	All (*n* = 182)	142		91	127	80	133	85	0.35
	Prostin (*n* = 92)	148		111	119	76	128	89	0.42
	Propess (*n* = 90)	138		75	138	84	138	80	0.81
		Average		SD	Average	SD	Average	SD	
PROM—Delivery (min)	All (*n* = 182)	1495		664	1152	505	1276	590	0.0001
	Prostin (*n* = 92)	1612		597	1155	508	1299	575	0.0001
	Propess (*n* = 90)	1402		707	1148	508	1253	607	0.07
		Average		SD	Average	SD	Average	SD	
Induction—Delivery (min)	All (*n* = 182)	1013		550	691	391	808	479	<0.0001
	Prostin (*n* = 92)	1114		478	680	348	817	440	<0.0002
	Propess (*n* = 90)	934		595	705	439	799	519	0.051

**Table 7:** Correlation between the individual delivery intervals.

Parameter	Values
PROM Induction vs. Induction Active phase interval	
Spearman (rho)	0.050
*p*	0.490
PROM Induction vs. Active phase Delivery interval	
Spearman	0.076
*p*	0.306
PROM Induction vs. PROM Delivery interval	
Spearman	0.076
*p*	0.31
PROM Induction vs. Induction delivery interval	
Spearman	0.07
*p*	0.37
Induction Active phase vs. PROM Delivery interval	
Spearman	0.83
*p*	<0.0001
Induction Active phase vs. Induction Delivery interval	
Spearman	0.98
*p*	<0.0001

Spearman—Spearman correlation coefficient. *p*—statistical significance.

**Table 8:** Logistic and linear regression analysis.

(a) Logistic regressions analysis for risk of operative delivery.				
**Predictor Factor**	**Regression Coefficient (Beta)**	**Significance Level (*p*)**		**aOR = Exp (Beta)**
Constant	−3.12	<0.001		0.04
Bishop Score	−0.42	0.256		0.66
Primiparous women	1.76	0.005		5.81
Induction Delivery Interval (ID interval)	0.0002	0.737		1.00
PROM—Delivery Interval (PD interval)	0.0003	0.0552		1.00
(b) Linear regression analysis for induction to delivery interval				
**Adjusted R-Squared: 0.976, *p* < 0.0001**				
**Predictor Factor**	**Coefficient**	**SE**	***t*** **Value**	***p*** **Value**
Intercept	57.82	17.38	3.33	0.001
Induction—Active phase interval	1.02	0.01	77.6	<0.001
Propess	9.38	11.11	0.84	0.399
Bishop score	0.6	12.22	0.05	0.956
Primiparous women	81.61	12.14	6.72	<0.001

## Data Availability

The data presented in this study are available on request from the corresponding author.
